# Quantification of Bisphenol A in the Saliva of Patients Wearing Clear Aligners

**DOI:** 10.3390/dj13120599

**Published:** 2025-12-15

**Authors:** Andrea Vitores-Calero, Verónica García-Sanz, Vanessa Paredes-Gallardo, Natalia Zamora-Martínez, Beatriz Tarazona-Álvarez

**Affiliations:** Department of Orthodontics, Faculty of Medicine and Dentistry, University of Valencia, 46010 Valencia, Spain; vitores@alumni.uv.es (A.V.-C.); veronica.garcia-sanz@uv.es (V.G.-S.); vanessa.paredes@uv.es (V.P.-G.); beatriz.tarazona@uv.es (B.T.-Á.)

**Keywords:** BPA, endocrine disruptor, aligners, composite

## Abstract

**Background/Objectives:** To analyze the behavior and release of bisphenol A (BPA) in the saliva of patients wearing clear aligners, and to evaluate differences in BPA levels between patients treated with aligners from the market-leading brand and those treated with in-office aligners. For the in-office group, 0.762 mm (0.30”) thick thermoforming sheets from Ortolan^®^ were used to fabricate the aligners. **Methods:** Patients about to begin orthodontic treatment with clear aligners in the Master’s degree program in Orthodontics at the University of Valencia were recruited for this prospective observational study. Patients were divided into two groups based on the type of aligners: The Invisalign^®^ group and the In-Office aligner group. Four saliva samples were taken from each patient at different times during treatment, with a one-week follow-up. The samples were stored at −80 °C and analyzed using mass spectrometry. **Results:** A total of 24 patients were included in the study, with 12 patients in each group. A statistically significant difference was found between baseline BPA levels and the increase observed half an hour after bonding the attachments. After a week, values returned to pre-treatment levels. Furthermore, BPA levels changed significantly during the follow-up period and were similar in both groups. **Conclusions:** An immediate increase was observed when the attachments were bonded with both treatments; however, differentiation from the ‘peak’ and recovery to baseline values was faster in patients treated with In-Office aligners. In those treated with Invisalign^®^, after the placement of the aligners, values recorded were not significantly different from baseline, nor from the previous peak.

## 1. Introduction

Bisphenol A (BPA) is a high-volume chemical primarily used in the production of polycarbonate plastics and epoxy resins. These are used to make food and drink containers, and their degradation is the main source of daily human exposure to BPA [1]. Due to its molecular structure, which closely resembles that of the natural hormone estrogen (17β-estradiol), BPA can exert teratogenic effects even at low doses. It acts as an endocrine disruptor by mimicking the structure of endogenous oestrogens and interfering with hormonal signaling pathways. Consequences include the acceleration of puberty onset, feminisation in men, and carcinogenic effects on the breast and prostate [2].

The presence of BPA in saliva, urine and blood has been reported following the use of resin-based dental restorative materials. Nevertheless, these levels typically remain below the tolerable daily intake (TDI) of 50 µg/kg/day established by the United States Environmental Protection Agency (EPA) [3].

However, there is no consensus regarding the relationship between BPA levels and potential effects. This is because BPA’s biological activity is established at concentrations very close to the detection threshold of most standard analytical techniques [4,5].

In dentistry, the organic matrix of adhesive systems often contains bisphenol A glycidyl methacrylate (Bis-GMA), a monomer that can break down into BPA. This raises concerns about its potential cytotoxicity and systemic effects because its cytotoxicity and mutagenic properties have been confirmed in tissue cultures [6]. Beyond dental composites, BPA is also used in the production of thermoplastic orthodontic aligners. The degradation of these materials in the oral environment due to temperature and pH fluctuations, mechanical wear and enzymatic activity from bacteria or saliva can result in BPA being released into the saliva [7,8,9,10].

In recent years, the use of clear thermoplastic materials in orthodontics has increased significantly, driven by demand for more aesthetically pleasing and comfortable treatment options among adults [11] and preadolescents and children [12]. However, orthodontic treatment with clear aligners requires the placement of multiple, often bulky, composite resin attachments to enhance the clinical performance of the aligners [13,14,15].

Therefore, the main objective of this work was to analyze the presence of BPA in the saliva of patients wearing clear aligners. It is essential to ensure that the material used for the aligners and the composite attachments does not pose any health risks to patients.

### Hypothesis

It is hypothesized that aligners, in conjunction with attachments, increase BPA levels in patients’ saliva without exceeding the limits reported by international protection agencies.

## 2. Materials and Methods

### 2.1. Study Design, Participants, Sample Size and Ethical Considerations

This research is a prospective observational study, conducted on patients undergoing treatment as part of the Master’s Programme in Orthodontic Specialisation at the University of Valencia.

The study received approval from the University of Valencia’s Committee for Experimental Research Ethics Involving Humans (protocol code 2579760) and Biosafety Committee of the Experimental Research Ethics Commission. The study complies with the fundamental principles set out in the Declaration of Helsinki and the Council of Europe’s Convention on Human Rights and Biomedicine, and adheres to Spanish legislation on biomedical research, the protection of personal data, and bioethics.

This prospective observational study was reported in accordance with the STROBE guidelines (von Elm et al., 2008) [16].

The sample consisted of:

- Patients beginning orthodontic treatment with clear aligners in the Master’s Program in Orthodontic Specialization at the University of Valencia.
- Patients with no existing composite restorations.
- Patients with no impairment of salivary secretion.
- Patients are not occupationally exposed to high levels of BPA.

Once the sample was selected, patients were allocated into two groups based on the type of aligner used for treatment.

Participants were assigned to the two treatment groups using simple randomisation. An Excel spreadsheet with a random number generator function was used to distribute each patient independently and equitably into one of the two groups (Invisalign^®^ (Align Technology, Inc., Tempe, AZ, USA) or In Office), thus ensuring that there was no bias in the assignment process.

For the In Office group, 0.762 mm (0.30″) thick thermoforming sheets from the company Ortolan^®^ (Ortolan Dental SLVictoria—Gasteiz, Spain) were used for the fabrication of the aligners. Both materials belong to the family of thermoplastic polyurethanes.

All samples received the same standardized amount of resin attachments, approximately 3 × 2 mm in size, which were polymerized using a curing light with the Smartlitefocus^®^ photopolymerization lamp (Dentsply Sirona, York, PA, USA) with identical power output and exposure time (20 s) in order to minimize variability in potential BPA release. Finally, in all patients, the burrs were removed with a round tungsten carbide bur.

The sample size power calculation was (Table 1):

A minimum of n = 12 patients will be needed to detect an immediate change in BPA count as reported by Kang (large effect size) [17] as statistically significant with 80% power.

The mean values used were 0.46 ± 1.70 mg/mL at baseline and 5.04 ± 5.73 mg/mL immediately postoperatively, following the article by Kang et al. (2016) [17].

Therefore, our calculation focused on detecting a change in BPA levels between two time points using a paired *t*-test.

The correlation between the levels at the two measurement times is assumed to be moderate (0.5) in the absence of further information.

From there, the software estimates the effect size (d = 0.89), the non-centrality parameter (delta = 3.11) (i.e., how much the Student’s t-distribution shifts under the alternative hypothesis with respect to the null hypothesis), the value of the t-statistic for that effect size, and finally the required sample size.

These values were used as the basis for the statistical calculations conducted with the G*Power 3.1.9.7 software.

**Figure 1:**
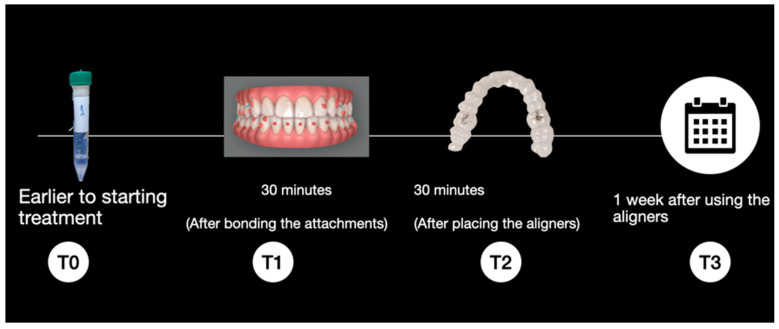
Sample collection timeline.

Sample Collection and Storage: Each patient was monitored over the course of one week, with saliva samples collected at the following time points (Figure 1):

- T0: Prior to the start of treatment without attachments or the aligner.
- T1: 30 min after bonding the attachments using the template with Transbond LR^®^ composite (3M Unitek, Saint Paul, Minnesota, United States).
- T2: 30 min after placement of the aligners, that is, 60 min after bonding the attachments.
- T3: After one week of aligner use

A systematic review was conducted on the subject and published (18). We found that the peaks of BPA release occurred 30 min after brackets were cemented or after the use of Essix or Hawley plates. Therefore, we wanted to record the release after 30 min of wearing the aligner and 30 min after the attachments were placed, which is when, according to the literature, the release is at its maximum. In addition, it was decided to take a sample once a week to see the BPA release when the aligner wear is at its peak, since they are changed weekly for a new one. On the other hand, we decided to obtain a sample of the patients before starting the research to see the baseline levels that each individual starts from, since it depends a lot on the habits of each one.

Participants should avoid eating, drinking (except water), smoking, chewing gum or brushing their teeth for at least 30 min before the sample was taken. They were also recommended not to consume alcohol or very fatty or spicy foods in the hours prior to the test, and to avoid intense exercise so as not to alter the components of the saliva.

The sample collection and storage protocol was as follows:

- While seated in the dental chair, each patient was given a paraffin pellet to stimulate salivary secretion, enabling the collection of approximately 1.5–2 mL of saliva. Using a disposable pipette, the saliva was transferred into plastic centrifuge tubes with screw caps.
- The samples were individually placed in sterilization pouches, labeled with numerical codes, and stored in a freezer at −80 °C.

The extraction hood used for sample processing was disinfected after each use and sterilized with ultraviolet light once a day. Furthermore, all containers were sterilized, and a new pipetting tip was used each time a sample was transferred from one container to another.

### 2.2. Sample Processing

Once a sufficient number of samples had been collected, they were removed from the −80 °C freezer approximately two hours prior to processing to allow them to thaw at room temperature.

After thawing, in the cell culture laboratory of the Central Research Unit (UCIM) at the University of Valencia, the samples were prepared in a room equipped with a fume hood. Personnel wore FFP2 masks, nitrile gloves, and protective goggles during handling.

Sample Processing Protocol:

1. One milliliter of each thawed sample was collected and transferred into a new test tube.
2. This milliliter of saliva was vortexed for 1 min.
3. After vortexing, 4.11 µL of 37% hydrochloric acid and 2 mL of methyl tert-butyl ether were added to the sample, followed by an additional minute of vortex agitation. This step was performed to ensure proper sample processing and to precipitate proteins, thereby facilitating subsequent analysis by mass spectrometry.
4. The mixture was then centrifuged at 3000 rpm for 5 min to separate the components.
5. Then, 1 mL of the supernatant was collected into an Eppendorf tube and evaporated in an Eppendorf Concentrator 5301 at 60 °C for 10 or more minutes, depending on the sample, until the liquid was completely evaporated.
6. The dry residues were reconstituted in 50 microliters of methanol and injected into a preparation plate for analysis using high-performance liquid chromatography coupled with mass spectrometry (HPLC-MS) at the Jerónimo Muñoz research building laboratory on the Burjassot Campus of the University of Valencia.

The BPA concentration data, expressed in parts per billion (ppb), from the analyzed samples were recorded in an Excel spreadsheet and subjected to statistical analysis.

### 2.3. Statistical Analysis

The statistical analysis was performed with st Halley Statistics^®^ software. The descriptive analysis provides the most relevant statistics for each parameter and difference: mean, standard deviation, minimum, maximum, median, and quartiles. The information is segmented by the ‘aligner type’ cross-variable.

Since the subgroups of aligner types have small sample sizes and some unusually high BPA values are detected, the analytical approach will be non-parametric. Therefore, the descriptive statistics will be reported in terms of median and interquartile range (IQR).

The inferential analysis aims to determine whether there are significant differences in BPA levels over time and based on the type of aligner. To do this, a non-parametric Brunner-Langer model for longitudinal data is estimated, with the within-subject factor being time (T0, T1, T2, T3) and the between-subject factor being the group or aligner type. The ATS statistic from the model will be considered for main effects and interactions.

Intra-group (time effect) multiple comparisons are established using the Wilcoxon test, and between-group (group effect) comparisons are made with the Mann-Whitney test. In both cases, the Bonferroni correction was applied.

The significance level used in the analysis was 5% (α = 0.05).

## 3. Results

### Relevant Descriptive Data

The final sample consisted of 24 patients eligible for orthodontic treatment with aligners, who were divided into two groups based on the aligner system they were going to use. On one hand, we have the Invisalign^®^ group with a total of 12 patients, and on the other hand, the In-Office group with another 12 patients (Table 2).

In the overall sample, a median of 6.94 ppb (IQR: 4.66–10.6) was recorded at baseline, which increased to 84.9 ppb (28.1–130.9) 30 min after the attachment bonding and then decreased to 10.1 ppb (5.39–14.6) after 30 min of placing the aligner in the mouth. Finally, these levels remained stable at 10.3 ppb (4.04–24.5) one week after the procedure. In the In Office group, a median of 9.26 ppb (IQR: 6.44–14.9) was recorded at baseline, which increased to 107.0 ppb (45.2–220) half an hour after the attachment bonding, and decreased to 9.52 ppb (7.75–14.6) after the aligner was placed in the mouth. Finally, these levels remained stable at 12.6 ppb (6.76–22.0) one week after treatment.

**Figure 2:**
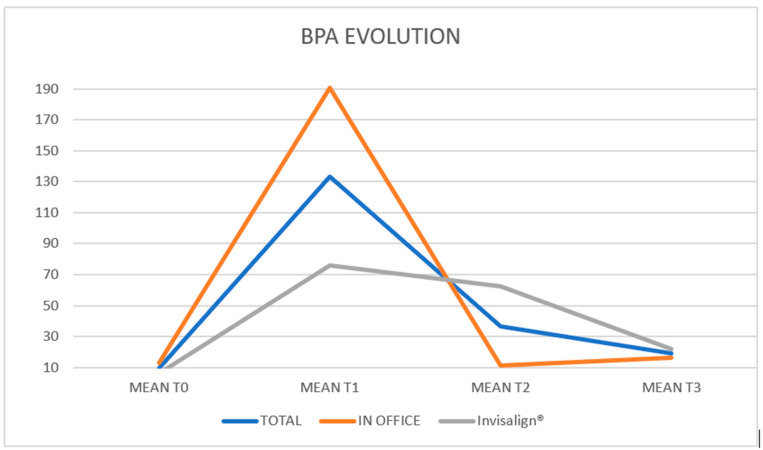
Representation of the variations in the BPA means at T0, T1, T2 and T3 of the total sample and of each group (In-office and Invisalign^®^).

In the Invisalign^®^ group, a median of 5.03 ppb (IQR: 2.27–7.0) was recorded at baseline, which increased to 37.3 ppb (10.5–127.3) half an hour after the attachment bonding, and decreased to 11.6 ppb (3.17–14.3) after the aligner was placed in the mouth. Finally, these levels remained stable at 7.09 ppb (2.93–30.4) one week after treatment (Figure 2).

Descriptive analysis and raw values of BPA levels in T0, T1, T2 and T3 for each group are provided in Supplementary Tables S1 and S2 to facilitate reproducibility.

The descriptive impression is that BPA levels are higher at one week than at baseline. Regarding group differences, BPA levels are higher in the In Office group; however, these levels are already elevated at baseline, so the difference cannot be attributed to the aligner type. The statistical model will address these issues.

The post-attachment measurement (T1) is significantly higher than any other. However, after the aligners were placed, there were no differences compared to baseline levels (*p* = 0.456), and after one week, the values were also at pre-treatment levels (*p* = 0.660) (Table 3).

Table 4 shows the changes in BPA levels throughout the follow-up by group:

- Overall, BPA changes significantly during the follow-up period (*p* < 0.001). This variation should be considered similar in both groups (*p* = 0.545).
- Reciprocally, BPA measurements are significantly different between the two groups (*p* = 0.001); but the differences remain at all time points (*p* = 0.545).

**Table 4:** Changes in BPA over the follow-up period by Group: Results of the ATS test from the Brunner-Langer model (*p*-value) for main effects and interaction.

	*p*-Value
Time	<0.001 ***
Group	0.001 **
Time × Group	0.545

*** *p* < 0.001; ** *p* ≤ 0.01;.

A few interesting nuances emerged (Table 5):

- In the In Office group, the increase at T1 is followed by an immediate return to baseline levels, which are maintained until the weekly visit.
- In the Invisalign^®^ group, the increase at T1 is followed by a more moderate decrease, with levels returning to a point that is not significantly different from the T1 peak or the baseline.

**Table 5:** Changes in BPA over the follow-up period by Group: Results of intra-group multiple comparisons (time effect) with the ATS test from the Brunner–Langer model and Bonferroni correction:.

	In Office	Invisalign^®^
T0 vs. T1	0.024 *	0.048 *
T0 vs. T2	1	0.300
T0 vs. T3	1	0.816
T1 vs. T2	0.018 *	1
T1 vs. T3	0.012 *	0.360
T2 vs. T3	1	1

* *p* < 0.05.

Therefore, just before the start of treatment, patients treated with In-Office aligners had a significantly higher BPA level (*p* = 0.048). At any other later time, the values had homogenized (Table 6).

## 4. Discussion

After conducting a systematic review on the topic and publishing our findings in the Journal of Clinical and Experimental Dentistry [18], we observed that several articles in the literature have demonstrated the presence of BPA in saliva, urine and blood following the use of adhesives and composites. As in our study, we found a statistically significant increase in BPA in patients’ saliva.

Firstly, Stocker et al. (2024) [19] studied BPA release in 11 patients before and after attachment removal. This is the first and only article we found in the literature that studies BPA in vivo in patients wearing aligners. Compared to the present study, two main differences emerge: the sample size is almost double, and a one-week follow-up of the patients was included.

Other studies, such as those by Manoj et al. (2018) [20] and Kang et al. (2011) [17], included a one-month follow-up of patients with metal brackets. The study by Watanabe et al. (2004) [21] examined BPA release from polycarbonate brackets and had a much longer patient follow-up period of 40 months.

In the present observational study, sample collection after one month was omitted because the aligners are changed weekly, meaning the point of maximum plastic degradation occurs just before changing to a new aligner, i.e., after one week of use. However, in line with the studies by Moreira et al. (2016) [3], Manoj et al. (2018) [20] and Kang et al. (2011) [17], a sample was collected 30 min after the procedure, as they found that maximum BPA release occurred 30 min after bracket bonding.

In this observational study, sample processing followed the methodology used by Kang et al. (2011) [17] in their study, in which they analyzed samples using high-resolution liquid chromatography coupled with mass spectrometry. The same approach was taken in this research and in the studies by Manoj et al. (2018) [20], Görükmez et al. (2021) [6] and Stocker et al. (2024) [19]. In contrast, Moreira et al. (2016) [3] and Kloukos et al. (2015) [22] employed gas chromatography coupled with mass spectrometry. Finally, Watanabe et al. (2004) [21] employed scanning electron microscopy.

BPA is sensitive to several factors, including masticatory stress, pH fluctuations, enzymatic degradation and temperature changes; higher temperatures lead to greater BPA release [15]. In line with the methodology used in the study by Kang et al. (2011) [17] and the study by Raghavan et al. (2017) [2], we decided to store the samples at −80 °C.

Other studies [3,22], which stored the samples at 37 °C and 4 °C, respectively, used gas chromatography coupled with mass spectrometry. In contrast, Görükmez et al. (2021) [6] and Stocker et al. (2024) [19] stored their samples at −20 °C.

In the present study, we observed a significant increase in BPA levels after bonding the attachments, which then decreased. This may simply be due to the fact that these levels decrease when rinsed for the first time with water or some other liquid, as we found in the study of Görükmez et al. (2021) [6].

Although BPA was detected in saliva after attachment bonding, saliva does not reflect systemic exposure, as BPA is metabolised in the liver and intestine and is mainly eliminated in urine; of approximately 1 L of saliva produced daily, around 10 µg of BPA would be found in it, but less than 1% is actually absorbed into the bloodstream. Thus, the estimated exposure levels remain far below the tolerable daily intake (TDI) established by the U.S. Environmental Protection Agency (50 µg/kg/day) [3]. According to the updated European Food Safety Authority threshold (0.2 ng/kg/day) [23], in which authorities have significantly lowered the maximum intake limit allowed because they have observed that BPA is very harmful to the body at very low doses and, in addition, there are many ways to easily ingest BPA on a daily basis, previous studies estimate that total oral exposure to BPA from dental procedures can range from 0.2 ng to 533 ng per treatment, with serum concentrations calculated in children between 10^−6^ and 10^−3^ nM, which translates to less than 10% of the daily TDI established by the EFSA (0.2 ng/kg/day) for a 20 kg child [24].

The EFSA acknowledges that this value is extremely restrictive and based on chronic exposure models, so it does not necessarily imply a real clinical risk in a specific dental context. 

Regarding the limitations of this study, comparisons with other studies were difficult to perform, as the majority were conducted with brackets and each study used a different methodology and sample processing method for analysis, as well as different units of measurement for BPA. Also, the processing of the samples must be very careful so that all samples have the same amount of reagents. Additionally, we did not include a prior questionnaire to learn about the patients’ habits or the use of plastic containers.

Due to this great diversity, it was challenging to faithfully reproduce the methodology of any one study, and 10 samples were lost at the beginning of the study, because we tried to evaporate the samples using another methodology described in the literature, but it did not work because the machine vaporized the samples instead of evaporating them. This could have been due to the volume of the final sample, the temperature used, or some other detail not specified in the article. On the other hand, the complex sample processing led to the loss of 10 samples with any slight change or modification in the process. Furthermore, if we had collected more samples at different times and extended the follow-up, we could have observed the actual fluctuation of BPA more closely.

By conducting a prior systematic review, we were able to identify errors in other studies and thus avoid them. For example, store the samples at an appropriate temperature, process the samples correctly to remove salivary proteins prior to spectrophotometric analysis, take the samples with a follow-up period, and do not let the patient rinse after bonding the attachments, so as not to decrease the actual concentration of BPA. Furthermore, since there are various methodologies and sample processing methods, we were able to select the one we considered most appropriate for performing a BPA analysis based on the findings obtained and what we observed in the literature, since the method used in this study to analyze BPA is considered the most reliable for detecting BPA levels.

The reasons why sample collection with directly printed aligners was not carried out in this study are the following: About halfway through the study, we acquired all the necessary machines for post-processing the aligners once they came out of the printer—that is, for curing and subsequent washing. However, when it came to acquiring the resin, it was not yet open to the general public because any minor error in post-processing could make the aligners toxic to humans. Therefore, only a company in Madrid, Spain, could safely print these aligners. We decided to wait until we could print the aligners ourselves to avoid any bias that might exist between printing in other laboratories, storage, and transportation. Furthermore, since this is a completely different material from that studied in this research, it could generate some bias in the methodology. Therefore, we are fully convinced that sharing these results with the scientific community can help other colleagues to advance in this field of research.

Our team is also preparing new research and collecting new data, as part of our future research with more brands, materials and improvements, such as a longer follow-up period and a larger sample size.

## 5. Conclusions

Overall, BPA salivary levels significantly increased after the placement of the attachments, returning to pre-treatment levels after the placement of the aligners and remaining stable until the weekly control visit, describing a pattern.

The immediate increase is observable with both treatments; however, the differentiation of the ‘peak’ and return to baseline values is faster with the In-Office group. In those treated with Invisalign^®^, after the placement of the aligners, values recorded were not significantly different from baseline, nor from the previous peak.

BPA levels are generally higher with In-Office aligners, even at baseline, so they cannot be attributed to a treatment effect. In fact, given the pattern described in the previous conclusion, significant differences are only detected before the attachments. Afterward, the records have equalized.

Based on the results of this study, we can confirm that attachments are another factor to add to the list of sources of BPA release, but much more research is needed in this field to truly determine the long-term safety of using this type of device.

## Figures and Tables

**Table 1:** Required sample size based on the relevant change in BPA quantity and statistical power (paired *t*-test). Calculated for a 95% confidence level and standard deviation based on the reference article [17].

Average BPA Count Values	Effect Size (d)	Power Achieved		
		70%	80%	90%
0.46–1.50	0.2 (small)	151	192	256
0.46–3.04	0.5 (medium)	27	33	44
0.46–5.04	0.89 (large)	10	12	16

**Table 2:** Descriptive analysis of the sample. N = number of patients; % = total percentage and percentage of patients in each group.

	N	%
Total	24	100.0%
In Office	12	50.0%
Invisalign^®^	12	50.0%

**Table 3:** Changes in BPA over the follow-up period: Results of multiple comparisons (time effect) with the ATS test from the Brunner–Langer model and Bonferroni correction.

	Global
T0 vs. T1	<0.001 ***
T0 vs. T2	0.456
T0 vs. T3	0.660
T1 vs. T2	0.024 *
T1 vs. T3	<0.001 ***
T2 vs. T3	1

*** *p* < 0.001; * *p* < 0.05.

**Table 6:** Changes in BPA over the follow-up period by Group: Results of inter-group multiple comparisons (group effect) with the Mann–Whitney test and Bonferroni correction.

	T0	T1	T2	T3
Group	0.048 *	0.640	1	1

* *p* < 0.05.

## Data Availability

The original contributions presented in this study are included in the article. Further inquiries can be directed to the corresponding author.

## Supplementary Materials

The following supporting information can be downloaded at: https://www.mdpi.com/article/10.3390/dj13120599/s1, Table S1: Descriptive analysis (mean, standard deviation, 25% and 75% percentiles, median) of BPA levels in T0, T1, T2 and T3 with In office aligners and Invisalign^®^; Table S2: Descriptive analysis (mean, standard deviation, 25% and 75% percentiles, median) of BPA levels between times T1-T0, T2-T0, T3-T0, T2-T1, T3-T1 and T3-T2 with In office aligners and Invisalign^®^.

## Abbreviations

The following abbreviations are used in this manuscript:

BPA	Bisphenol A
