# Supplementary material for: Quantification of Bisphenol A in the Saliva of Patients Wearing Clear Aligners
Source: Dent J (Basel). 2025 Dec 15;13(12):599. doi: 10.3390/dj13120599 (PMC12731839; doi:10.3390/dj13120599)
Supplement: Supplementary file 1 [file dentistry-13-00599-s001.zip › dentistry-3982920-supplementary.pdf]

Supplementary tables

Supplementary Table S1.- Descriptive analysis: BPA level in T0, T1, T2 and T3:

		GROUP		
		**Total**	**In office**	**Invisalign®**
**T0**	**N**	24	12	12
	**Mean**	9.31	12.75	5.86
	**Standard deviation**	8.73	10.46	4.91
	**Minimum**	1.70	3.00	1.70
	**Maximum**	40.48	40.48	19.71
	**25th percentile**	4.66	6.44	2.27
	**Median**	6.94	9.26	5.03
	**75th percentile**	10.59	14.88	7.00
**T1**	**N**	24	12	12
	**Mean**	133.13	190.42	75.83
	**Standard deviation**	185.53	241.43	80.74
	**Minimum**	6.72	8.53	6.72
	**Maximum**	834.00	834.00	258.00
	**25th percentile**	28.12	45.16	10.46
	**Median**	84.92	107.00	37.33
	**75th percentile**	130.95	220.00	127.30
**T2**	**N**	24	12	12
	**Mean**	36.62	11.01	62.23
	**Standard deviation**	91.65	5.74	126.89
	**Minimum**	1.77	2.61	1.77
	**Maximum**	340.60	23.84	340.60
	**25th percentile**	5.39	7.75	3.17
	**Median**	10.10	9.52	11.62
	**75th percentile**	14.61	14.61	14.32
T3	**N**	24	12	12
	**Mean**	19.08	16.18	21.98
	Standard deviation	24.61	12.16	33.16
	Minimum	1.07	2.69	1.07
	Maximum	116.90	39.75	116.90
	25th percentile	4.04	6.76	2.93
	Median	10.29	12.57	7.09
	75th percentile	24.50	22.03	30.40

Supplementary Table S2.- Descriptive analysis: BPA level between times:

		GROUP		
		Total	In office	Invisalign®
DIF.T 1_T0	N	24	12	12
	Mean	123.82	177.66	69.97
	Standard deviation	186.71	244.75	81.64
	Minimum	-31.95	-31.95	-8.85
	Maximum	826.08	826.08	249.97
	25th percentile	23.33	33.59	4.87
	Median	77.50	97.89	34.02
	75th percentile	123.52	207.07	123.52
DIF.T 2_T0	N	24	12	12
	Mean	27.31	-1.75	56.37
	Standard deviation	91.43	12.41	124.43
	Minimum	-31.86	-31.86	-3.73
	Maximum	324.17	12.26	324.17
	25th percentile	-1.69	-5.93	-0.65
	Median	3.21	2.86	3.42
	75th percentile	8.26	4.62	11.11
DIF.T 3_T0	N	24	12	12
	Mean	9.77	3.42	16.12
	Standard deviation	26.73	16.02	33.91
	Minimum	-32.12	-32.12	-15.66
	Maximum	111.56	31.83	111.56
	25th percentile	-2.73	-3.31	-2.37
	Median	1.67	-0.31	3.78
	75th percentile	17.60	15.13	25.37
DIF.T 2_T1	N	24	12	12
	Mean	-96.51	-179.41	-13.60
	Standard deviation	212.61	241.88	144.94
	Minimum	-823.93	-823.93	-246.00
	Maximum	329.74	0.09	329.74
	25th percentile	-120.65	-215.78	-103.26
	Median	-49.19	-98.42	-19.81
	75th percentile	-7.41	-25.68	4.59

DIF.T3_T1	**N**	24	12	12
	**Mean**	-114.05	-174.24	-53.85
	**Standard deviation**	188.08	237.11	99.18
	**Minimum**	-794.25	-794.25	-255.56
	**Maximum**	110.18	-0.17	110.18
	**25th percentile**	-124.57	-207.44	-124.57
	**Median**	-51.66	-86.61	-30.24
	**75th percentile**	-14.58	-36.57	6.09
DIF.T3_T2	**N**	24	12	12
	**Mean**	-17.54	5.17	-40.25
	**Standard deviation**	99.97	14.71	139.83
	**Minimum**	-336.55	-18.68	-336.55
	**Maximum**	112.80	29.68	112.80
	**25th percentile**	-4.70	-2.33	-7.92
	**Median**	0.71	2.12	0.71
	**75th percentile**	15.97	15.05	16.99
